# Make-up of the Dental Profession in Great Britain

**Published:** 1905-08

**Authors:** 


### Domestic Correspondence.

### MAKE-UP OF THE DENTAL PROFESSION IN GREAT BRITAIN.

"The *Dental Register* for 1905 has recently been issued, and shows that at last the number of those holding licenses in Dental Surgery exceeds that of those who appear therein by virtue of being in *bona fide* practice at the passing of the Act. It is true that the *Register* does not tell us exactly how many dentists are in practice, for there are some holding the license who have not subscribed their names. On the other hand, it is probable that many who did register at the passing of the Act are not in active practice, and that some others have definitely retired. The *Register*, however, constitutes the official record published for the General Medical Council, and contains the names of 4734 persons, an increase of fifty-eight over last year. Of these, 2374 held a license in dental surgery from one or other of the four colleges which grant such diplomas; of the rest, 2332 were admitted to the *Register* on the strength of the fact that they were in *bona fide* practice as dentists before the passing of the Dentists' Act of 1878; a few of these, however, held ordinary medical or surgical degrees or diplomas. The balance of the names in the *Register* is made up by three gentlemen admitted thereto as holders of Colonial dental certificates and by twenty-five others who have been granted degrees in dental medicine or dental surgery by recognized universities in the United States of America. Of the holders of these colonial or foreign diplomas, some ten or eleven appear to practise abroad. In regard to those who hold licenses in dental surgery, sixty per cent. of them derive their title from the Royal College of Surgeons of England, eighteen per cent. from that in Ireland, twelve per cent. from that in Edinburgh, and eight per cent. from the Faculty of Physicians and Surgeons of Glasgow. Of the total,

2374, some 280 hold ordinary medical and surgical degrees or diplomas in addition to one in dentistry."

The above, an editorial in the *British Journal of Dental Science* for May 15, 1905, page 458, shows conclusively that the provisions in the dental Act, to that end and the construction placed upon them by the General Medical Council, has proved a gratifying success in building up the home schools; of the two thousand four hundred and two admitted to the *Register* by virtue of an educational qualification, twenty-eight only obtained this qualification abroad. As a result of the encouragement the home schools have thus received, they have vastly improved, they have increased in number, and the well-trained practitioners they have year by year sent out have been a grand uplift to the profession. The profession and the community in Great Britain is now reaping the benefit of the wisely planned dental reform of nearly a generation ago.

